# Newt Opportunities for Understanding the Dedifferentiation Process

**DOI:** 10.1100/tsw.2006.327

**Published:** 2006-07-07

**Authors:** Ziad Y. Chaar, Catherine Tsilfidis

**Affiliations:** University of Ottawa Eye Institute Ottawa Health Research Institute, Ottawa, Ont. K1H 8L6, Canada

**Keywords:** newt forelimb regeneration, Notophthalmus viridescens, dedifferentiation, newt regeneration extract

## Abstract

Urodele amphibians, such as the newt *Notophthalmus viridescens*, have the unique ability to regenerate limbs, spinal cord, eye structures, and many vital organs through a process called epimorphic regeneration. Although the cellular basis of regeneration has been studied in detail, we know relatively little about the molecular controls of the process. This review provides an overview of forelimb regeneration in the newt, addressing what we know about cellular and molecular aspects. Particular focus is placed on the dedifferentiation process, which yields a population of embryonic-like pluripotent cells that will eventually reform the lost structure. This cellular plasticity seems to be the key to regenerative ability. We discuss the dedifferentiation process in newt forelimb regeneration and outline the various studies that have revealed that mammalian cells also have the ability to dedifferentiate if given the appropriate triggers.

## INTRODUCTION

The ability of adult organisms to undergo regeneration or repair is present to some extent in most multicellular phyla. However, the degree of regeneration complexity varies from one organism to another and from one tissue to another. The simplest form of tissue regeneration is highlighted in mammals by the axonal outgrowth of a severed peripheral nerve, repair of epidermal cuts, or healing of broken bones. A more complex form of regeneration involves hyperplasia in organs such as the liver, intestine, or adrenal gland. However, the most complex form of regeneration is the replacement of entire tissues and organs that are morphologically and histologically indistinguishable from the original. This is known as epimorphic regeneration and is mainly observed within the urodele amphibians, the newts and axolotls[1,2,3,4].

## CELLULAR BASIS OF EPIMORPHIC REGENERATION

Since it was first described by Spallanzani in 1768[5], the ability of urodele amphibians, such as the newt *Notophthalmus viridescens*, to regenerate amputated or injured structures has fascinated developmental biologists. This ability extends beyond limbs to include eye structures (lens and neural retina), cardiac muscle, tail, and spinal cord. More than 200 years after it was first described, we still know surprisingly little about the molecular mechanisms that control the epimorphic regeneration process. However, the cellular basis of regeneration is a little better understood.

Within minutes following fore- and hindlimb amputation, the bleeding is arrested by the formation of a fibrin clot (see Fig. 1). Soon thereafter, epidermal cells detach from the basement membrane at the edge of the wound surface and migrate across the fibrin network; by approximately 9 h postamputation, the wound is covered by a 3- to 4-cell-layer thick wound epithelium (WE)[6,7]. In subsequent regeneration stages, this WE thickens to form the apical epithelial cap (AEC), a structure that is functionally analogous to the apical epidermal ridge (AER) in mammalian limb development[8].

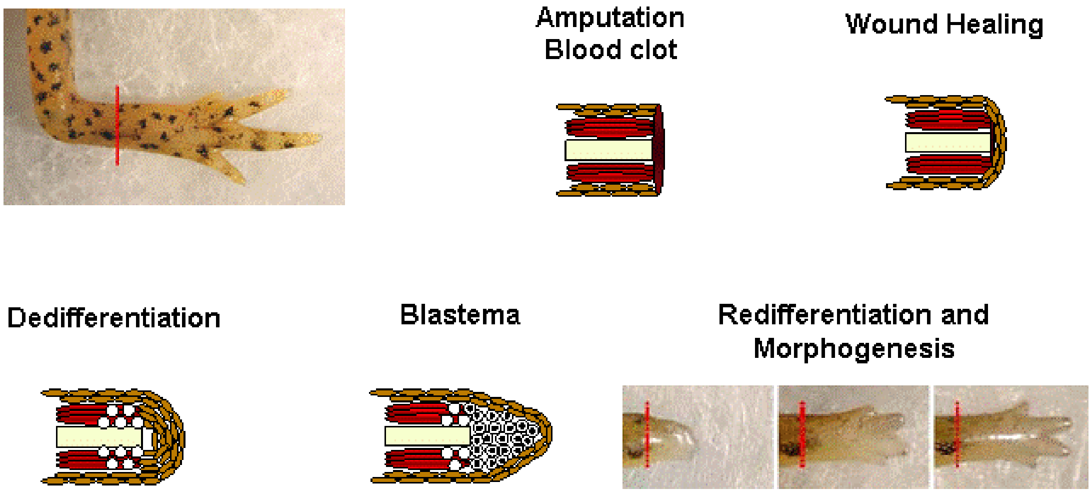

**FIGURE 1.** Stages of newt forelimb regeneration. On amputation of the newt forelimb, a fibrin clot is formed and epithelial cells migrate to cover the wound. Dedifferentiation ensues and is accompanied by a thickening of the wound epithelium to form the AEC. The multipotent cells that are generated by the dedifferentiation process then proliferate to form the blastema. During subsequent stages, blastema cells redifferentiate, resulting in the regeneration of the lost forelimb.

, amputation site; , blood clot; , epithelial cell; , muscle myotube; , bone; , dedifferentiating cell; , multipotent blastema cell.

Epithelial wound healing is followed by the onset of dedifferentiation, a process whereby fully differentiated cells just proximal to the amputation surface lose their specialized cytoplasmic characteristics and revert to a morphologically less specialized or “embryonic-like” state[9,10,11]. Morphological changes associated with dedifferentiation include rounded nuclei with prominent nucleoli, increased nuclear-cytoplasmic ratios, and increased numbers of free ribosomes[11,12]. Evidence for dedifferentiation has been provided by a number of studies, both cellular and molecular, showing the conversion of one differentiated cell type to another during the regeneration process and will be further discussed below[13,14,15,16,17,18,19,20,21].

Following dedifferentiation, the dedifferentiated cells accumulate at the apex of the regenerate and proliferate to form a mesenchymal mass, termed the blastema. As the blastema is forming at the apex of the regenerate, dedifferentiation at the base continues to contribute to the blastema population. In addition, further contributions to the regenerating cell population may be provided by resident stem cells; thus, the blastema may arise from a combination of dedifferentiation and stem cell activation[22,23,24,25].

A key feature for the formation of the blastema is the presence of a thickened AEC[26]. The formation of the basement membrane beneath the WE is postponed until the blastema has been established, allowing direct contact of the AEC with the underlying mesenchymal cells[27]. It has been postulated that the AEC provides a “division signal” that causes successive rounds of replication in the blastema cells and prevents precocious differentiation until a critical mass of cells is attained[28,29,30]. A second key requirement for blastema formation is the presence of an adequate nerve supply[31]. Innervation provides neurotrophic factors for proliferation of blastema cells. The role of nerves in epimorphic regeneration has been extensively reviewed[31,32,33,34,35].

In subsequent regeneration stages, the limb blastema continues to grow and transforms from a small bud into an elongated cone and eventually a flattened palette. These changes are indicative of the onset of redifferentiation and morphogenesis. Redifferentiation progresses as a wave from the base of the blastema towards its distal tip. Cartilage condensation and myogenesis are initiated first and are followed by the differentiation of connective tissue, the blood vascular system, as well as the establishment of nervous connections[36]. The progression of these morphogenetic events and the continuous growth of the regenerate result in a perfectly functional replacement. Factors such as temperature and nutritional intake influence the rate of the regeneration process, but typically, histogenesis is complete within approximately 2 months. Thereafter, the overall size of the limb and the pigmentation pattern may continue to develop, so that by the time that regeneration is complete, the limb is indistinguishable from its contralateral counterpart.

## MOLECULAR BASIS OF EPIMORPHIC REGENERATION

While the cellular basis of regeneration has been extensively studied, molecular insights have only started to emerge. An exhaustive analysis of all the identified genes is beyond the scope of this review, and so we will deal with a select few at the various stages of the regeneration process. Interestingly, many of these genes are not only expressed during the regeneration process, but also during the initial development of the limb, prompting the theory that regeneration is a recapitulation of development. However, there are enough examples of differences in gene profiles between development and regeneration to challenge the complete validity of this theory.

During wound healing and the generation of the WE in urodeles, fibroblast growth factors (FGFs) and their receptors (FGFRs) appear to be essential[37,38,39]. FGF-2 is found in the AEC and in nerves. Denervation results in the dramatic down-regulation of protein levels in both tissues and causes arrest of regeneration. Exogenously applied FGF-2 restores regenerative potential[39]. Interestingly, in *Xenopus laevis*, a similar mechanism appears to be conserved. FGFR-1 and -2 are expressed in the WE at premetamorphic stages that are permissive to regeneration, but expression is lost concomitant with the loss of regenerative potential in postmetamorphic stages[40]. Furthermore, inhibitors of FGFR-1 and -2 abrogate regenerative ability in premetamorphic stages[40], showing the essential role of these genes in the progression of regeneration. Similarly, FGF-8 and -10 are expressed in the developing limb bud epidermis and mesenchyme, respectively, (reviewed by Martin[41]) as well as in the regenerating *Xenopus* blastema[42], but their expression is lost in the nonregenerating postmetamorphic stages. Furthermore, exogenously applied FGF-10 stimulates regeneration of a nonregenerating limb, suggesting a key role for FGF-10 in limb regeneration[43].

During the dedifferentiation stage of limb regeneration, genes that are implicated in extracellular matrix (ECM) degradation and remodeling are essential. Members of the matrix metalloproteinase (MMP) family are key players during this stage of regeneration. MMP activity has been shown to be up-regulated during early stages of limb regeneration[44,45,46], leading to the degradation of ECM and the destabilization of the differentiated state. Recently, Vinarsky et al. have demonstrated that a further potential function for MMPs is to prevent scar tissue formation during regeneration[46]. MMPs have been shown to be involved in cell proliferation and migration in mammalian systems, and may have similar roles in limb regeneration. Additional extracellular proteases, such as thrombin and elastase, have also been implicated in dedifferentiation and extracellular remodeling[47,48,49].

Vascotto et al. conducted representational difference analysis (RDA) in order to identify genes associated with the dedifferentiation process. This screen isolated cDNAs from several gene classes, including putative transcription factors, immune modulators, proteases, and genes involved in cytoskeletal remodeling[50]. The homeobox gene *msx-1* has also been shown to have a key role in dedifferentiation[51,52] and will be discussed further below.

There are a large number of genes that cannot clearly be assigned to a particular stage of regeneration because their expression profiles and influence span more than one or two regeneration stages. Some of the above mentioned *fgfs* and *MMPs* fall into this category. Additional examples are members of the *Hox* family, *retinoic acid receptors (RARs)*, *sonic hedgehog* (*shh*), and *Tbox* genes. All of these genes have been implicated in limb development in other species, but clearly have roles in the urodele regeneration process as well.

The Hox transcription factors are key regulators in the outgrowth and pattern formation of the developing limb, and appear to be involved in specifying positional identity. *Hox* genes are re-expressed during urodele forelimb regeneration and may be similarly involved in specification of proximo-distal characteristics[53]. Retinoic acid (RA) is also involved in the specification of positional memory. Treatment of a regenerating limb with RA leads to proximalization of positional information and duplication of structures[54,55]. Thus, a limb amputated through the radius-ulna behaves as though it was amputated through the elbow or the humerus. The degree of proximalization depends on the length and concentration of the RA treatment. RA exerts its effects through RARs. RAR isoforms appear to play different roles in the regenerate varying from growth inhibition to proximo-distal respecification[54,56,57,58]. *shh* is another gene shown to have a key role in determining positional information. s*hh* expression was first identified as the signal in the zone of polarizing activity (ZPA) that was responsible for antero-posterior (AP) patterning of the chicken limb bud[59]. In the newt regenerate, *shh* expression is seen in the mesenchymal cells in the posterior region of the blastema where it also appears to control AP identity, suggesting that a ZPA also exists in the regenerating limb[60,61]. *Tbox* genes determine limb identity in higher vertebrates. *Tbox-5* is exclusively expressed in forelimbs and *Tbox-4* is exclusively expressed in hindlimbs[62,63,64,65]. Similar profiles are seen during hindlimb and forelimb regeneration in the newt[66,67].

Since similar genes are often expressed during both development and regeneration, this has led to the conclusion that regeneration is a recapitulation of development. However, it is important to note that gene expression profiles are not always conserved between the two processes. For example, *Tbox-4* and *-5* are expressed in both hindlimb and forelimb during embryonic newt development[66,67], but follow the mammalian patterns of restricted expression during the regeneration process. *HoxC6* shows different patterns of expression during newt limb development and regeneration[68,69,70], as do some members of the *HoxA* cluster[71]. Furthermore, developing and regenerating limbs do not appear morphologically similar. The initial patterning of the developing limb involves the sequential appearance of digits 1 and 2, followed by digits 3 and 4[72]. During regeneration, all the digits seem to appear at once[73].

## THE DEDIFFERENTIATION PROCESS: KEY TO REGENERATIVE ABILITY

Normally, once cells have completed the differentiation pathway, they are terminally committed. The dedifferentiation process in newt forelimb regeneration is a notable exception to this general rule. In the forelimb, dedifferentiation of mature tissues just proximal to the amputation surface (within 1–2 mm[74]) yields the source of multipotent, progenitor-like cells that will eventually repopulate the limb. Cells of the mature stump tissues are freed from their intercellular matrices, undergo morphological changes, and acquire embryonic-like characteristics[11,12].

A number of studies have provided conclusive evidence for dedifferentiation. For example, when multinucleated myotubes containing a lineage tracer are reimplanted into a regenerating limb, labelled mononuclear cells appear in the regenerate and are seen in the differentiating cartilage[17,18]. *In vivo* studies in the axolotl have shown that labelled myotubes in a regenerating tail break up to give rise to mononucleate cells that provide a considerable contribution to the formation of the blastema[15]. In addition, cells can switch fate from one lineage type to another, as shown by the conversion of ectoderm derived radial glial cells to muscle and cartilage in the regenerating tail[16].

Dedifferentiation not only occurs *in vivo*, but can also be triggered *in vitro*. Newt myotubes can be stimulated to undergo cell cycle re-entry in culture in response to serum. Myotubes transverse the S-phase of the cycle and arrest at G2. In mammals, this “reversion” in muscle differentiation and re-entry into the cell cycle is blocked by hypophosphorylation of the retinoblastoma (Rb) protein, making mammalian myotubes refractory to serum stimulation. However, myotubes isolated from Rb homozygous null (Rb -/-) mice are able to transverse the S-phase of the cell cycle in response to serum stimulation[75], suggesting that Rb regulation is key to cell cycle re-entry during dedifferentiation. This is confirmed by studies showing that serum resupplementation of newt myotubes leads to Rb phosphorylation, a process that does not occur in mammalian myotubes[76]. The serum factor that promotes cell cycle re-entry appears to be downstream of thrombin activation, since purified thrombin, in the presence of subthreshold levels of serum, can induce S-phase re-entry[47,48]. However, it is clear that dedifferentiation requires more than one factor since, in the presence of thrombin and serum, myotubes are not able to complete the cell cycle and fragment into mononucleate cells, as they do *in vivo.*

## MAMMALIAN REGENERATIVE CAPABILITY

Examples of remarkable regenerative ability are extremely rare in mammals. Limited regenerative ability has been shown in embryonic or fetal tissues, but this ability is severely curtailed in the adult organism. Notable exceptions are provided by the regeneration of deer antlers[77], fingertips in children and mice[78,79] and ear holes and heart tissue in the MRL mouse[80].

It has been suggested that regenerative ability is an ancient attribute that has been lost with time and evolution, and there have been several theories that have been advanced to explain why such a potentially advantageous trait might be selected against. One theory suggests that the production and maintenance of a pluripotential population of blastema cells may predispose an organism to the formation of tumors[81,82]. Paradoxically, newts have been shown to have a marked resistance to tumor formation in epimorphic regenerating fields[83,84,85,86]. Thus, the same mechanism that initiates a regeneration response in urodeles (i.e., cell cycle re-entry), leads to apoptosis in mammals[1]. A second theory suggests that since epimorphic regeneration is a lengthy process, it is more efficient for mammals to initiate an efficient, rapid wound healing process involving scar formation[82,87]. Immune cells and cytokines are central players in the formation of this scar and ultimately interfere with the regeneration response, suggesting that immunodeficiency may be related to regenerative ability (reviewed by Harty et al.[88]). A third, and altogether different possibility, is that no selective pressure existed (either positive or negative) during evolution to maintain or eliminate regenerative ability[81, 82], and so this trait has been lost in most higher vertebrates.

Regardless of the reasons for loss of regenerative ability, the absence of dedifferentiation in higher vertebrates is the most probable reason why regenerative ability is limited. Thus, an understanding of the basic mechanisms involved in the dedifferentiation process could have tremendous clinical applications in the treatment of injury or degenerative disease. Studies suggest that mammalian cells do have the ability to dedifferentiate if provided the appropriate triggers (see Fig. 2). When interspecies hybrid myotubes (heterokaryons) are formed by the fusion of newt with mouse C2C12 myogenic cells, the C2C12 nuclei within these heterokaryons respond to serum stimulation by re-entering the cell cycle, whereas C2C12 nuclei within homokaryons are incapable of cell cycle re-entry[89]. Clearly, the newt cells are able to provide a key factor (or factors) that makes the mammalian cells competent to respond to serum stimulation, and allows them to initiate the dedifferentiation response.

McGann et al. showed that the dedifferentiation factors are present in regenerating newt limb extracts. Following treatment with these extracts, both newt and mouse myotubes not only transverse the S-phase of the cell cycle, but are also able to fragment into mononucleated cells. This dedifferentiation is accompanied by the down-regulation of myogenic proteins such as MyoD, myogenin, and troponin T [90].

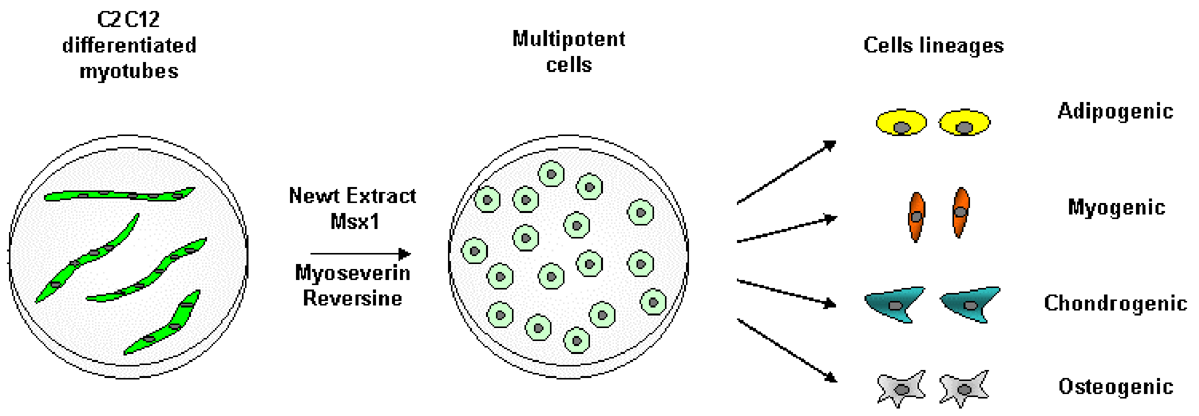

**FIGURE 2.** C2C12 myotubes dedifferentiate into multipotent progenitor cells. Differentiated C2C12 myotubes undergo dedifferentiation and fragment into mononucleate cells in response to newt extract[83], *msx1* expression[42], myoseverin[91], or reversine[92]. The mononucleated cells generated are multipotent and were shown to redifferentiate into cells expressing adipogenic, myogenic, chondrogenic, or osteogenic markers[42,91,92].

The identification of the dedifferentiation factors is being actively pursued by a number of laboratories. The homeobox gene, *msx1*, is one possible candidate[51,52]. *Msx1* was previously shown to delineate the border between undifferentiated (*msx1*-expressing) and differentiating (non-*msx1*-expressing) cells in the developing mammalian limb bud[91,92]. Furthermore, ectopic expression of *msx1* in cultured mouse cells was able to inhibit myogenesis[93,94]. In regeneration studies, Simon et al. showed that *Msx1* expression is found in the early newt regenerate, during the dedifferentiation phase of regeneration [52]. Additional studies have shown that *msx1* is required for regenerative processes in several different species. In *X. laevis* tadpoles, *msx1* can promote spinal cord regeneration during the refractory period between stages 45 and 47[95]. In fetal and neonatal mouse digit tip regeneration, *msx1* is an important regulator of the regeneration response[96,97]. In cultured salamander myotubes, the use of morpholino antisense oligonucleotides directed against *msx1* blocks the dedifferentiation and fragmentation of myotubes into mononucleate cells[98]. All these studies suggest that msx1 is an important regulator of regenerative potential. Interestingly, ectopic expression of *msx1* coupled with serum stimulation can induce C2C12 mouse myotubes to dedifferentiate and fragment into mononucleated cells *in vitro*[99]. This dedifferentiated cell population can then be induced to redifferentiate into cells expressing chondrogenic, adipogenic, myogenic or osteogenic markers[99].

The screening of small compound chemical libraries has provided a different approach for the identification of dedifferentiation inducing molecules. These studies have led to the identification of two purine compounds, myoseverin[100] and reversine[101], which are capable of inducing dedifferentiation. Myoseverin is a microtubule-binding trisubstituted purine that can induce C2C12 myotube dedifferentiation and cleavage into mononucleated cells. Its effects are likely related to cytoskeletal remodeling. Reversine, a disubstituted purine compound, is able to induce C2C12 myotubes to dedifferentiate into multipotent cells, which can then be induced to differentiate into osteogenic and adipogenic lineages[101].

Compounds such as myoseverin and reversine, and genes such as *msx1*, have offered tremendous insights into the dedifferentiation process and suggest that there may be multiple ways to induce the dedifferentiation pathway. These studies, combined with others aimed at identifying the dedifferentiation factors, will generate important tools that researchers will be able to use to enhance regenerative ability in mammals.

## SUMMARY

Animals such as the newt have much to teach us about the regulatory control of regenerative ability. At a site of injury, newt cells are able to undergo a reversal in cell fate to yield a population of multipotent progenitors that can then redifferentiate and transdifferentiate to form all the tissue of the regenerating structure. It is clear that this dedifferentiation process is key to regenerative ability.

Although we still have considerable gaps in our understanding of the epimorphic regeneration process, researchers are beginning to unravel some of the molecular pathways that control regenerative ability. By understanding the regulatory mechanisms that control epimorphic regeneration in newts, and in particular the dedifferentiation process, we may be able to apply this knowledge to improve regenerative capabilities in humans.

## ACKNOWLEDGMENTS

Due to the overwhelming number of publications in the regeneration field, we apologize for the inadvertent omission of key references. This work was supported by grants from the Natural Sciences and Engineering Council of Canada (NSERC) and the Canadian Institutes of Health Research (CIHR).
